# Omega-3 fatty acids and L-carnitine prevent meiotic oocyte damage induced by follicular fluid from infertile women with endometriosis: an experimental study

**DOI:** 10.5935/1518-0557.20230023

**Published:** 2023

**Authors:** Vanessa Silvestre Innocenti Giorgi, Rui Alberto Ferriani, Paula Andrea Navarro

**Affiliations:** 1 Human Reproduction Division, Department of Gynaecology and Obstetrics, Faculty of Medicine of Ribeirao Preto, University of Sao Paulo, Ribeirao Preto, SP, Brazil; 2 National Institute of Hormones and Women’s Health, National Council for Scientific and Technological Development (CNPq), Brazil

**Keywords:** endometriosis, female infertility, follicular fluid, oocyte quality, omega-3 fatty acids, L-carnitine

## Abstract

**Objective:**

To assess whether follicular fluid (FF) from infertile women with endometriosis in advanced stages [moderate/severe (EIII/IV) without or with endometrioma (Endometrioma)] induce more oocyte damages than in early stages (minimal/mild: EI/II); and whether supplementation with L-carnitine (LC) and omega 3 (n3) can prevent these oocyte damages.

**Methods:**

Experimental study using bovine oocytes (obtained of ovaries from slaughterhouse), and human FF (samples were obtained during oocyte recovery for ICSI). Bovine oocytes were submitted to *in vitro* maturation (IVM) divided into 9 groups: no FF(No-FF), with 1% FF from infertile women without endometriosis (FFC), with EI/II, EIII/IV and Endometrioma, and with (or not) LC+n3 addition. After IVM, oocytes were fluorescently labelled and visualized by confocal microscopy to analyze chromosomes and spindle.

**Results:**

FF from endometriosis decreased rate of normal MII (spindle assembly and chromosome alignment) compared to No-FF (87.2%) and FFC (87.2%). FFEIII/IV (80.7%) and FFEndometrioma (69.3%) decreased total MII rate compared to No-FF (91.9%) and FFC (89.2%), and FFEndometrioma had lower total MII rate compared to other groups. LC+n3 increased MII rate in the FFEIII/IV (80.7% *vs*. 90.8%) and the Endometrioma (69.3% *vs*. 86.4%), and it prevented damages in spindle and chromosomes in MII oocytes in the FFEI/II group (62.2% *vs*. 84.5%) and the FFEIII/IV group (70.2% *vs*. 84.1%).

**Conclusions:**

FF of endometriosis damaged the meiotic spindle of bovine MII oocytes. EIII/IV led to impaired nuclear maturation; FF from women with endometrioma had further negative impact in oocyte maturation. LC+n3 completely prevented the effects of FF from women with endometriosis on oocyte.

## Introduction

Endometriosis is defined as the presence and development of endometrial cells outside the uterine cavity (Kennedy *et al*., 2005). For women with infertility, the prevalence of endometriosis is 25-50% and, for women with endometriosis diagnosis, approximately 30-50% them have reduced fertility (Missmer *et al*., 2004).

The American Society of Reproductive Medicine (ASRM) classifies endometriosis as: minimal (I), mild (II), moderate (III) and severe (IV) (ASRM, 1997). Most women with endometriosis III/IV have pelvic anatomical alterations that impede ovulation and tubal transport of the embryo (de Ziegler *et al*., 2010). However, women with endometriosis I/II, in which there are no anatomical alterations of the pelvic cavity, also have decreased fertility rates (Bérubé *et al*., 1998; Parazzini, 1999).

The decrease of oocyte quality in women with endometriosis contributes to infertility (Mansour *et al*., 2010; Da Broi *et al*., 2014; Barcelos *et al*., 2009) but the mechanisms responsible for this effect are unknown (Simón *et al*., 1994; Díaz *et al*., 2000). Addition of follicular fluid (FF) from infertile women with endometriosis stages I/II into an *in vitro* maturation (IVM) medium of bovine oocytes reduces the quality of oocytes (Giorgi *et al*., 2016) and embryos in vitro produced from these oocytes (Giorgi *et al*., 2021). The antioxidants L-carnitine (LC) and N-acetyl-cysteine (NAC) protect against meiotic damages to these oocytes, suggesting that oxidative stress (OS) has a role in the etiopathogenesis of infertility and endometriosis (Giorgi *et al.*, 2016; 2021).

LC also functions as part of transport of long chain fatty acids from the cytosol into the mitochondria for β-oxidation (Evans & Fornasini, 2003). β-oxidation is essential for the resumption of oocyte meiosis and nuclear maturation in mice (Downs *et al*., 2009; Paczkowski *et al*., 2013; Valsangkar & Downs, 2013), swine and cattle (Paczkowski *et al*., 2013).

Previous studies evaluated the addition of omega-3 fatty acids into the IVM medium of bovine oocytes (Oseikria *et al*., 2016; Nikoloff *et al*., 2017). Oseikria *et al*. (2016) showed that addition of low concentrations of docosahexaenoic acid (DHA) increased oocyte competence as indicated by increased cleavage rates and blastocyst formation after parthenogenetic activation (Oseikria *et al*., 2016). Nikoloff *et al*. (2017) showed that the addition of eicosapentaenoic acid (EPA) improved the oocyte quality, as indicated by increased *cumulus* cell expansion.

No studies have yet evaluated the impact of the endometriosis stage on oocyte quality, and the possible prevention of oocyte damage by the combined administration of LC, DHA and EPA. Therefore, we used confocal microscopy to compare the impact of FF from different groups of women (controls, with endometriosis I/II, III/IV without endometrioma and III/IV with endometrioma) to the IVM medium of bovine oocytes on nuclear maturation and organization of the meiotic spindle and chromosomes. We then examined the impact of LC and DHA/EPA(n3) on the amelioration of FF-induced oocyte damage.

## Materials and Methods

This study was approved by the Research Ethics Committee of the Clinic Hospital of the Medical School of Ribeirão Preto (HC-FMRP), University of São Paulo (USP) (Process HCRP nº 12201/2008) and by the Ethics Committee in Animal Experimentation of FMRP - USP (nº 169/2008).

### Patient selection and FF collection

FF samples acquired from March to October of 2013 and from May 2016 to September 2017 were from infertile women who underwent ovarian hyperstimulation for intracytoplasmic sperm injection (ICSI) in the Sector of Human Reproduction, Department of Gynecology and Obstetrics, Faculty of Medicine of Ribeirão Preto, University of São Paulo (FMRP-USP) (eligible patients). All patients provided written informed consent prior to participation.

All women with endometriosis had the following characteristics: younger than 40 years; body mass index (BMI) of 30kg/m^2^ or less; serum concentration of follicle stimulating hormone (FSH) of 12 mIU/mL or less; free of chronic anovulation, hydrosalpinx, chronic diseases, endocrinopathy, cardiovascular conditions, and infection; non-smoking; not use of anti-inflammatory agents, hormonal medications, or vitamin complexes in the 6 months before treatment of assisted reproduction techniques (ART); and previous diagnosis of endometriosis based on videolaparoscopy. Women in the control group had the same characteristics and were infertile due to tubal and/or male factors.

Women with endometriosis were subdivided into 3 groups [early stage endometriosis (EI/II) and advanced endometriosis without (EIII/IV) or with an active endometrioma in the cycle visualized by transvaginal ultrasonography (Endometrioma)].

The Endometrioma group represented women with active lesion of endometriosis since all of endometriosis women underwent to treatment during videolaparoscopy.

Samples of FF obtained during 2013 were previously tested in experiments of bovine IVM for evaluate their potentiality to cause meiotic abnormalities. Results are presented in supplementary material.

### Controlled ovarian stimulation protocols

Previously to controlled ovarian stimulation (COS), all patients used combined oral contraceptive and in accordance with the characteristics of each patient, one of two COS protocols was chosen:

- Flexible antagonist protocol: gonadotrophins (150 to 300IU/day) administered daily on the first 6 days, with the dose adjusted daily according to follicular growth.

- Minimal stimulation protocol (clomiphene citrate plus gonadotrophins and a GnRH antagonist), in which clomiphene citrate (100 mg/day) was administered daily on the first 5 days and gonadotrophins (150 IU/day) was administered on days 2 and 4, and daily from day 6.

Administration of GnRH antagonist (ganirelix or cetrorelix 0.25mg/day) began when the mean diameter of the largest follicle was 14 mm or more. Recombinant hCG (250 µg, Ovidrel^®^, Serono, Brazil) or urinary hCG (10,000 IU, Choriomon^®^, Meizler, Brazil) was administered when at least one follicle had a diameter of 18 mm. Oocytes were collected 34 to 36 h after administration of recombinant hCG, and the luteal phase was maintained by micronized progesterone (600 mg/day).

### Collection and processing of FF samples

FF samples were obtained during oocyte recovery for ICSI. To prevent repetitive punctures, FF was only acquired from the first follicle (diameter ≥15mm) of the first punctured ovary.

In Endometrioma group, samples of FF were from ovary with (2/8) or without endometrioma (6/8).

Samples were immediately taken to the embryology laboratory, where embryologists checked for the presence of oocytes and/or granulosa cells. Oocytes were separated from the FF for use during ART. The FF was centrifuged at 300 *g* for 10 min, aliquoted, and stored at -80^o^C. All FF samples without oocyte and/or granulosa cells, and samples contaminated with blood were discarded.

### Bovine oocyte collection

The ovaries of cows were collected after slaughter and transferred into physiological saline at 35 to 38.5^o^C. In the laboratory, follicles with diameters of 2 to 8 mm were aspirated, and *cumulus*-oocyte complexes (COCs) with uniform cytoplasm and three or more layers of cumulus oophorus cells were selected (Adona & Lima Verde Leal, 2004; Ferreira *et al*., 2009).

### *In vitro* maturation

COCs (about 20 per drop) were cultivated without mineral oil in TCM-199 containing Earle’s salts and bicarbonate (Invitrogen, Gibco Laboratories Life Technologies, Inc., Grand Island, NY, USA) supplemented with 0.4mM sodium pyruvate, 0.5µg/mL gentamicin, 5µg/mL FSH, 2.5 UI/mL hCG (Chorulon^®^), 1 µg/mL estradiol and 10% foetal calf serum (FCS; Gibco); at 38.5°C, 95% humidity and 5% CO_2_ (Hashimoto *et al.* 2002; Adona & Lima Verde Leal, 2004; Ferreira *et al*., 2009). The duration of IVM was 22-24 h.

### Concentration of follicular fluid on IVM medium

The concentration of FF added to IVM medium was 1% based on previous study of Da Broi *et al*. (2014) that tested different concentrations of FF from infertile women with and without mild endometriosis on medium of IVM of bovine oocytes. The concentrations of FF tested were 1%, 5%, 10% and 15%, and no dose-response was observed (Da Broi *et al*., 2014). So, we used the lowest tested concentration (1%).

### L-carnitine and omega-3 solutions

The concentration of LC (Sigma Aldrich C0283) in the IVM medium was 0.6 mg/mL (Mansour *et al*., 2009; Giorgi *et al.*, 2016), and LC was stored in a 100 x stock solution that was prepared with water, filtered (0.22 µm), aliquoted, and stored at -20°C prior to use.

The concentration of omega-3 fatty acids in the IVM medium was 1 nM [0.4nM was DHA (Sigma Aldrich D2534) and 0.6 nM EPA (Sigma Aldrich E2011)] (Nikoloff *et al*., 2017). The ratio of 2:3 DHA:EPA was chosen based on previous randomized clinical trials (Nadjarzadeh *et al*., 2015; Haghiac *et al*., 2015; Rahbar *et al.*, 2012). A stock solution (100x) was prepared using DMSO, filtered (0.22µm), aliquoted, and stored at -20°C.

### Immunofluorescence of bovine oocytes and visualization of microtubules and chromosomes

After 22-24h of IVM, *cumulus* cells were removed by pipetting, and the oocytes were fixed in a buffer for microtubule stabilization (Liu *et al*., 1998; Ferreira *et al*., 2009). The oocytes were then washed and blocked in washing medium [phosphate buffer saline (PBS) with 0.02% NaN_3_, 0.01% Triton X-100, 0.2% defatted dry milk, 2% goat serum, 2% bovine serum albumin, and 0.1 M glycine] for 2 h at 37ºC. Incubation with an anti-β-tubulin murine monoclonal antibody (1:1000) was performed overnight at 4^o^C. After washing, a secondary fluorescein isothiocyanate (FITC)-conjugated anti-mouse IgG antibody (1:500; Zymed Laboratories, Invitrogen, Carlsbad, CA, USA) was added at 38.5^o^C for 2 h. The oocytes were washed again and labelled with Hoechst 33342 (10 mg/mL) in Vectashield mounting medium (H-1000, Vector, Burlingame, CA, USA), placed on a glass slide, and covered with a coverslip.

A confocal microscope (Confocal Leica TCS SP5, Leica Microsystems, Mannheim, Germany) with 405 nm diode UV and a 543 nm HeNe laser was used to visualize the oocytes at 40 x.

### Oocyte classification

First, oocytes were classified according to nuclear maturation, as being in metaphase I (MI), telophase I (TI), metaphase II (MII), or undergoing parthenogenetic activation (PA). MII oocytes were categorized based on metaphasic plate visualization, as analyzable (meiotic spindle in lateral or sagittal position) or non-analyzable (meiotic spindle in polar position) (Ju *et al.*, 2005).

**Figure 1:**
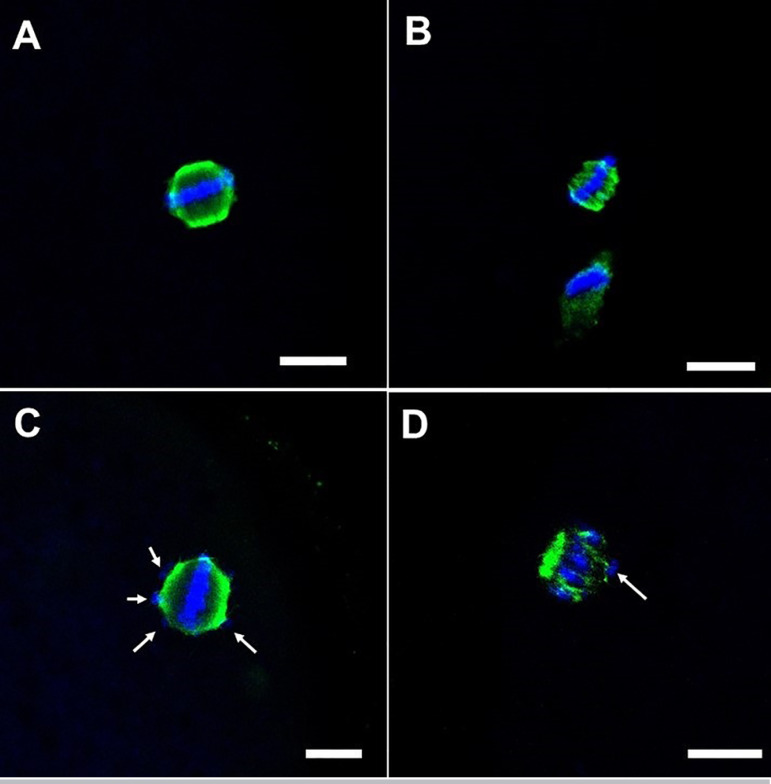
Representative confocal microscopy images (40x) of bovine oocytes matured *in vitro* during metaphase II, based on organization of the meiotic spindle and chromosomal alignment. Normal MII: A; Abnormal MII: B, C, D. *Note: Scale bar: 10 µm; White arrows: misaligned chromosomes*.

MII oocytes observed in lateral/sagittal position were considered: “normal” when meiotic spindle had typical barrel shape and chromosomes arranged in line at the equator of the spindle (Figure 1D); and as “abnormal” when meiotic spindle had reduced size and was disarranged or dispersed from the plane of the metaphasic plate (Figure 1A-C). PA was defined as the presence of two polar bodies or the presence of telophase II.

### Statistical analysis

Data were analyzed using R Studio software version 1.0.153 (https://www.R-project.org).

Clinical variables, response to COS, and ICSI results of women donors of FF were compared between the four groups (control, EI/II, EIII/IV and Endometrioma) using Kruskall Wallis test with Dunn post test.

The categorical variables (rates of MI, TI, PA, MII, MII analyzable, normal MII) were compared between the 9 groups using the Chi-square test.

For all comparisons a *p* value below 0.05 was considered.

## Results

Eight IVM experiments were performed, and one FF sample of each group was used individually in each experiment.

### Flow-chart of selection of FF donors

**Figure 2:**
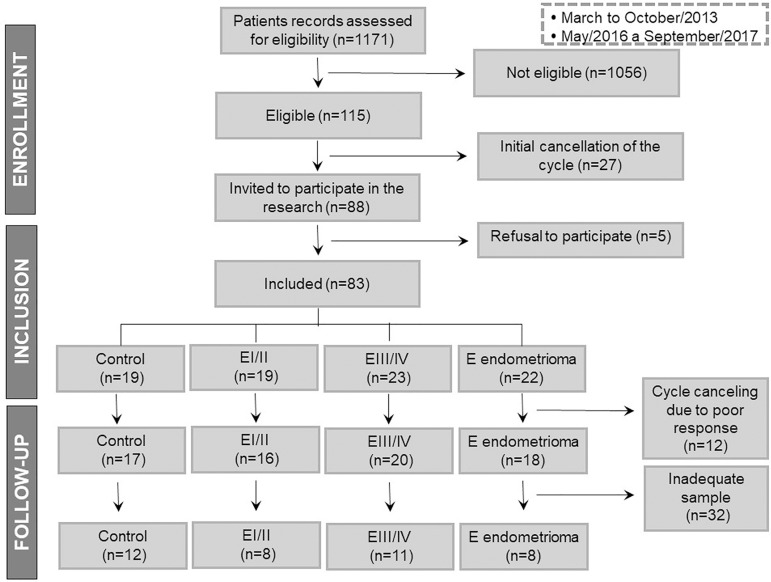
Flow-chart of recruitment of donors of follicular fluid.

Figure 2 represents Flow-chart of selection of FF donors.

A total of 39 FF samples were processed and stored at -196°C until use. The choice of samples in each experiment was chosen based on age, BMI and COS protocol. In Control and EIII/IV groups, only FF samples from women with BMI between 19 and 29 were included. Table 1 shows characteristics of FF donors used in each group.

**Table 1:** Clinical variables, response to controlled ovarian stimulation, and ICSI results of infertile women with no endometriosis (control), women with endometriosis I/II (EI/II), women with endometriosis III/IV without endometrioma (EIII/IV) and women with endometriosis III/IV with endometrioma (Endometrioma).

	Control (n=8)	El/ll (n=8)	E lll/IV (n=8)	E endometrioma (n=8)	
Age (years)	36 (35.5 - 37.3)	34 (33.5 - 37)	33 (29 - 34)	34.5(33-36.3)	*p* =0.1907
BMI (kg/m^2^)	24.6 (23.8 - 27.9)	21.9 (21.8-27.4)	24.8 (24.6 - 27.3)	21.8 (20.8 - 25.2)	*p* =0.3259
FSH (Ul/L)	8.2 (7.9 - 9.7)	4.4 (4.4 - 5.3)	5.2 (3.8 - 7.8)	5.6 (4.1 - 8.4)	*p* = 0.1890
Duration of infertility (months)	114(93-135)^*^	84 (81 - 84)	108 (66-111)	48 (45 - 60)^*^	^*^p = 0.0275
Time vlpsc and COS (months)	57 (53.8 - 70.5)	23 (32.8 - 75)	68 (10.3-79)	34 (30 - 49.5)	*p* = 0.3282
Basal CFA (n)	8.0 (7.5-8)	7 (7-9.5)	9.0 (3.8-11)	8.0 (3-13)	*p* = 0.9533
Endometrium (mm)	3.3 (2.9 - 3.9)	7.3 (2.9 - 5.5)	4.6 (3.2 - 6.9)	4.5 (3.4 - 5.2)	*p* = 0.6850
COS duration (dias)	8 (8 - 9.3)	9(9-10.5)	10(9-10)	10(9.5-11.3)	*p* = 0.2115
Recovery oocytes (n)	3(2-4)	4 (3.8 - 5.5)	4 (3.5 - 8)	2 (1 - 4.3)	*p* = 0.1873
Mature oocytes (n)	3 (2 - 3.3)	4 (3.8 - 5.5)	4 (3.5 - 7.5)	2 (1 - 4.3)	*p* = 0.2084

Note: The variables are reported as median and interquartile ranges. BMI: body mass index; FSH: follicle stimulating hormone; CFA: count of antral follicles; COS: controlled ovarian stimulation; vlpsc: videolaparoscopy. Compared groups by Kruskall Wallis test with Dunn post test (*p* < 0.05).

### *In vitro* maturation and confocal microscopy

During the 8 IVM experiments (performed between November 2017 and January 2018), 1686 immature COCs were submitted to IVM, 1561 oocytes were fixed for immunofluorescence, and 1401 oocytes were visualized by confocal microscopy. A total of 167 oocytes were in MI, 25 were in TI, 1188 in were in MII, and 21 were PA. Among the 1188 oocytes in MII, 735 were analyzable.

The 9 experimental groups had no differences in TI (*p*=0.05467), PA (*p*=0.8854), and analyzable MII (*p*=0.5651) (Table 2).

**Table 2:** Stages of nuclear maturation, and percentages of normal MII oocytes matured in vitro in medium without follicular fluid (No-FF), with addition of 1% FF from infertile patients without endometriosis (FFC), with early endometriosis (FFEI/II), with advanced endometriosis without (FFEIII/IV) or with endometrioma (FFEndometrioma), supplemented with 0.6 mg/mL L-carnitine and 1 nM omega-3 (LC+n3) visualized by confocal microscopy.

	Fixed oocytes after IVM n	Viewed by confocal N	MI n (%)	TI n (%)	PA n (%)	MII		
						Total MII n (%)	Analyzable n (%)	Normal n (%)
No-FF	167	149	9(6.0%)^a^	2 (1.3%)	1 (0.7%)	137 (91.9%)^a^	94 (68.6%)	82 (87.2%)^a^
FFC	163	148	9 (6.1%)^a^	5 (3.4%)	2 (1.4%)	132 (89.2%)^ab^	78 (59.1%)	68 (87.2%)^a^
FFC+LC+n3	179	163	19 (11.7%)^ab^	3 (1.8%)	3 (1.8%)	138 (89.2%)^ab^	80 (58.0%)	66 (82.5%)^ab^
FFEI/II	178	158	20 (12.7%)^ab^	1 (0.6%)	2 (1.3%)	135 (85.4%)^ab^	82 (60.7%)	51 (62.2%)^c^
FFEI/II +LC+n3	186	163	16 (9.8%)^ab^	4 (2.5%)	4 (2.5%)	139 (85.3%)^ab^	84 (60.4%)	71 (84.5%)^ad^
FFEIII/IV	185	166	28 (16.9%)^bc^	2 (1.2%)	2 (1.2%)	134 (80.7%)^b^	84 (62.7%)	59 (70.2%)^bc^
FFEIII/IV +LC+n3	163	142	11 (7.7%)^a^	1 (0.7%)	1 (0.7%)	129 (90.8%)^a^	82 (63.6%)	69 (84.1%)^ad^
FFEndometrioma	163	150	37 (24.7%)^c^	7 (4.7%)	2 (1.3%)	104 (69.3%)^c^	66 (63.5%)	48 (72.7%)^bcd^
FFEndometrioma +LC+n3	177	162	18 (11.1%)^ab^	0 (0%)	0 (0%) 4 (2.5%)	140 (86.4%)^ab^	85 (60.7%)	64 (75.3%)^abc^

Note: IVM: *in vitro* maturation; MI=metaphase I; TI=telophase I; MII=metaphase II; PA=parthenogenetic activation. Analyzable: oocytes with spindles fixed in lateral or sagittal view. Data are results of 8 replicates using follicular fluid from control patients, with mild endometriosis, with severe endometriosis without endometrioma and with endometrioma. Different letters in the same column indicate statistical difference, *p* <0.05, using chi-square test.

The rate of MI, in the No-FF group (6.0%) was similar to that of the FFC group (6.1%, *p*=1), the FFC+LC+n3 group (11.7%, *p*=0.1277), the FFEI/II group (12.7%, *p*=0.07406), the FFEI/II+LC+n3 group (9.8%, *p*=0.3085), the FFEIII/IV+LC+n3 group (7.7%, *p*=0.3085), and the FFEndometrioma+LC+n3 group (11.1%, *p*=0.166). In the other hand, the rate of MI was lower in the No-FF and FFC groups when compared to the FFEIII/IV (16.9%; *vs*. No-FF: *p*=0.00504; *vs.* FFC: *p*=0.00537) and FFEndometrioma (24.7%, *vs.* No-FF: *p*<0.0001, *vs.* FFC: *p*<0.0001). The addition of LC+n3 had no effect on the MI rate in the FFC group (6.1% *vs.* 11.7%, *p*=0.192) and FFEI/II group (12.7% *vs.* 9.8%, *p*=0.5288). However, the FFEIII/IV+LC+n3 group had a lower MI rate than the FFEIII/IV group (7.7% *vs.* 16.9%, *p*=0.0259), and the FFEndometrioma+LC+n3 group also had lower MI rate than FFEndometrioma group (11.1% *vs.* 24.7%, *p*=0.0028).

The total MII rate was 91.9% in the No-FF group, similar to the FFC group (89.2%, *p*=0.5389), the FFC+LC+n3 group (84.7%, *p*=0.06992), the FFEI/II group (85.4%, *p*=0.1069), the FFEI/II+LC+n3 group (85.3%, *p*=0.09598), the FFEIII/IV+LC+n3 group (90.8%, *p*=0.1069) and the FFEndometrioma+LC+n3 group (86.4%, *p*=0.1681). The lowest total MII rate was in the FFEndometrioma group (69.3%), and this was significantly lower than all other groups (*vs.* No-FF: *p*<0.0001; *vs.* FFC: *p*<0.0001; *vs.* FFC+LC+n3: *p*=0.00194, *vs.* FFEI/II: *p*=0.00114, *vs.* FFEI/II+LC+n3: *p*=0.00117, *vs.* FFEIII/IV: *p*=0.02681, *vs.* FFEIII/IV+LC+n3: *p*<0.0001; *vs.* FFEndometrioma+LC+n3: *p*=0.00044). The No-FF group had a higher rate of total MII than the FFEIII/IV group (80.7%, *p*=0.00681). The total MII rate was similar in the FFC group (89.2%) and the FFC+LC+n3 group (89.2%, *p*=0.3122), the total MII rate was also similar in the FFEI/II+LC+n3 group (85.3%) and the FFEI/II group (85.4%, *p*=1.0). However, the addition of LC+n3 increased the rate of total MII in the FFEIII/IV group and the FFEndometrioma group [(FFEIII/IV *vs*. FFEIII/IV+LC+n3: *p*=0.0190) and (FFEndometrioma *vs.* FFEndometrioma+LC+n3: *p*=0.0004)].

The percentage of normal MII was 87.2% in the No-FF group, similar to the FFC group (87.2%, *p*=1.0), the FFC+LC+n3 group (82.5%, *p*=0.54), the FFEI/II+LC+n3 group (84.5%, *p*=0.7615), the FFEIII/IV+LC+n3 group (84.1%, *p*=0.7122) and the FFEndometrioma+LC+n3 group (75.3%, *p*=0.0623). The percentage of normal MII was significantly greater in the No-FF group (87.2%) than in the FFEI/II group (62.2%, *p=*0.00023), the FFEIII/IV group (70.2%, *p*=0.0092) and the FFEndometrioma group (72.7%, *p*=0.03497). The FFC group also had a significantly higher percentage of normal MII than the FFEI/II group (*p*=0.00059), the FFEIII/IV group (*p*=0.01523) and the FFEndometrioma group (*p*=0.0486). The addition of LC+n3 during IVM did not alter the rate of normal MII in the FFC group (*p*=0.5792) nor in the FFEndometrioma group (*p*=0.865). However, LC+n3 increased the rate of normal MII in the FFEI/II group (*p*=0.00205) and the FFEIII/IV group (*p*=0.04995). Although the FFEndometrioma group (72.7%) and the FFEndometrioma+LC+n3 group (75.3%) had similar percentages of normal MII (*p*=0.865), the FFEndometrioma+LC+n3 group had a similar percentage of normal MII as the No-FF group (*p*=0.062) and the FFC group (*p*=0.083).

## Discussion

This study demonstrated that the FF from infertile women with any stage of endometriosis decreased the quality of bovine oocytes that were cultured in IVM medium. The FF of women with endometrioma had an even more detrimental impact on oocyte quality, in that this FF affected nuclear maturation and promoted meiotic abnormalities. The addition of LC+n3 prevented the oocyte damages induced by FF from women with endometriosis. These results suggest that OS and alterations in β-oxidation decrease oocyte quality during the early and advanced stages of endometriosis.

The results presented here confirm previous data of our group, which showed that addition of FF from infertile women with mild endometriosis to IVM medium of bovine oocytes led to damage of the meiotic spindle (Da Broi *et al*., 2014; Giorgi *et al*., 2016). A novel finding of the present study is that the FF of infertile women with advanced endometriosis also led to meiotic damage of bovine oocytes.

We observed an impairment of maturation rate of bovine oocytes in the presence of FF from women with endometriosis in stage III/IV with or without endometrioma. Hamdan *et al*. (2016) also observed that the FF from women with severe endometriosis impaired *in vitro* oocyte maturation. These authors suggested that the decreased polar body extrusion rate is a consequence of DNA damage caused by OS (Hamdan *et al*., 2016).

We demonstrated that the FF from infertile women with endometriosis III/IV with endometrioma had the greatest impact on nuclear maturation. A recent retrospective cohort study showed a lower number of quality embryos in the group of women with endometrioma compared to women without endometriosis, although the cumulative live birth rate did not differ between groups (Zeng *et al*., 2022). An endometrioma is characterized by an accumulation of iron and its derivatives, making the environment toxic and hostile to folliculogenesis (Sanchez *et al*., 2014). Analysis of FF by mass spectrometry indicated the presence of 535 expressed proteins, and that 139 of these proteins occurred in the FF of both ovaries of women with unilateral endometrioma and control women (Regiani *et al*., 2015), demonstrating the impact of endometriosis (or presence of an active endometrioma in the cycle, regardless of the follicular proximity) on the composition of FF (Regiani *et al.*, 2015). However, it would be interesting future studies assessing FF of both ovaries from women with unilateral endometrioma on oocyte quality and OS markers.

We found that LC+n3 prevented the oocyte damage induced by FF from women with endometriosis. Our previous study reported that LC protected against injuries to the meiotic spindle of *in vitro* bovine oocytes induced by FF from women with mild endometriosis (Giorgi *et al*., 2016). Thus, a novel finding of the present study is that LC+n3 together prevent oocyte damage.

A review highlighted the important role of carnitine in female fertility by analysis of *in vivo* and *in vitro* studies with humans and animal models, and described the possible mechanisms by which LC improves female fertility (Agarwal *et al.*, 2018). Experimental studies showed that supplementation with n3 improves oocyte quality (Nehra *et al*., 2012), regulates the endometrium (Waters *et al.*, 2014) and increases the pregnancy rate (Wathes *et al*., 2007). Also recent studies showed the importance of EPA/DHA rich dietary in composition of FF and in improve cleavage rate in patients of ART (Kermack *et al.*, 2020; 2021).

LC is an antioxidant that reduces OS and lipotoxicity by eliminating free radicals, and thereby decreases apoptosis and promotes oocyte growth and development (Agarwal *et al*., 2018). LC and fatty acids have roles in β-oxidation, an important energy production pathway during oocyte maturation (Downs *et al*., 2009; Paczkowski *et al*., 2013; Valsangkar & Downs, 2013; Dunning *et al.*, 2014). Oocytes from infertile women with endometriosis I/II have mitochondrial alterations, based on analysis by transmission electron microscopy and RT-PCR (Xu *et al*., 2015). Although we did not directly investigate oocyte mitochondria in this study, we suggest that the FF of women with endometriosis alters mitochondrial function, and thereby reduces oocyte quality.

Our evaluation of the clinical data of the different FF donor groups (FFC, FFEI/II, FFEIII/IV and FFEndometrioma) indicated a difference in the duration of infertility for the control group and the group with endometriosis III/IV with endometrioma. We believe this difference is not relevant to our outcome because these two groups had a similar median age (a factor strongly related to worsening oocyte quality). Another important point is that the FF samples were not paired by type of COS protocol, and in the literature the role of stimulation in oocyte quality is still controversial (Thaker *et al*., 2020; Montoya-Botero *et al*., 2021; Jirge *et al*., 2022); however, the number of days of stimulation and the amount of FSH used by women were similar comparing the groups.The present study helped to elucidate the etiopathogenesis of infertility due to endometriosis. However, there were some limitations. First, the sample size was small, limiting the generalizability of the results; however, we used rigorous criteria for selection of all FF donors. Second, we used FF of women who were submitted to COS, so extrapolation of our findings to women undergoing natural cycles is questionable; however, women in the control group were scheduled for COS, making our comparisons valid. Third, we used an *in vitro* bovine oocyte maturation assay, so direct extrapolation to humans is not possible. Studies with human oocytes matured *in vivo* are needed to corroborate our findings. And fourth, the technique used to analyze the oocytes (confocal microscopy) was limited to assessed oocytes fixed in polar position, resulting in 58%-68% of MII analyzable.

Our major findings were that FF from infertile women with endometriosis damages meiotic spindle assembly and alters chromosome alignment of MII bovine oocytes. The FF from women with endometriosis III/IV besides damage the meiotic spindle, also impairs nuclear maturation; and FF from women with endometrioma leads to additional impairment of nuclear maturation. Supplementation with LC+n3 prevented all these damaging effects.

The currently available treatments for infertility due to endometriosis are surgery and/or ART (Kennedy *et al*., 2005). These treatments are invasive and/or costly, and therefore unavailable to many people. New therapeutic approaches are needed to help many women whose infertility is due to endometriosis. Our findings suggest that clinical studies should investigate the impact of a combination of surgical treatment with LC+n3 supplementation for preventing the recurrence and/or progression of endometriosis and improving natural fertility.

## Supplementary Material

During January to March 2017, we performed 4 experiments to test samples of follicular fluid (FF) obtained in 2013 (storage at -196oC).

The experiments were similar as described in Materials and Methods of this paper. Therefore, we used immature bovine oocytes matured *in vitro* in medium with FF from infertile women without endometriosis, with early endometriosis (EI/II) or with advanced endometriosis (EIII/IV). All of FF samples were obtained in 2013.

After 22-24 h of *in vitro* maturation (IVM), the oocytes were denuded and fixed to immunofluorescence. Stage of nuclear maturation, spindle assembly and chromosome alignment were assessed by confocal microscopy. Table I shows the results.

During the 4 IVM, 534 immature cumulus-oocyte complexes were submitted to IVM, 509 oocytes were fixed for immunofluorescence, and 482 oocytes were visualized by confocal microscopy. A total of 102 oocytes were in metaphase I (MI), 7 were in telophase I (TI), 357 were in metaphase II (MII), and 16 were parthenogenetically activated (PA). Among the 357 oocytes in MII, 204 were analyzable.

The 4 experimental groups had no differences in rates of MI (*p*=0.3453), TI (*p*=0.5177), PA (*p*=0.5619), total MII (*p*=0.6511) and analyzable MII (*p*=0.8839) (Table I).

**Table 1:** Stages of nuclear maturation, and percentages of normal MII oocytes matured *in vitro* in medium without follicular fluid (No-FF), with addition of 1% FF from infertile patients without endometriosis (FFC), with early endometriosis (FFEI/II) and with advanced endometriosis (FFEIII/IV) visualized by confocal microscopy.

	Oocytes viewed by confocal n	MI n (%)	TI n (%)	PA n (%)	MII		
					Total MII n (%)	Analyzable n (%)	Normal n (%)
No-FF	101	20 (19.8%)	1 (1.0%)	4 (4.0%)	76 (75.2%)	46 (60.5%)	37 (80.4%)^a^
FFC	124	23 (18.7%)	1 (0.8%)	4 (3.3%)	96 (78.0%)	56 (58.3%)	45 (80.4%)^a^
FFEI/II	99	18 (18.2%)	3 (3.0%)	5 (5.1%)	73 (73.7%)	40 (54.8%)	23 (57.5%)^b^
FFEIII/IV	158	41 (25.9%)	2 (1.3%)	3 (1.9%)	112 (70.9%)	62 (55.4%)	41 (66.1%)^ab^

Note: MI=metaphase I; TI=telophase I; MII=metaphase II; PA=parthenogenetic activation. Analyzable: oocytes with spindles fixed in lateral or sagittal view. Data are results of 4 replicates using follicular fluid from control patients, with mild endometriosis and with severe endometriosis. Different letters in the same column indicate statistical difference, *p*<0.05, using chi-square test.

The percentage of normal MII was 80.4% in the No-FF group, similar to the FFC group (80.4%, p=1.0) and FFEIII/IV (66.1%, *p*=0.1544). The group FFEIII/IV (66.1%) also had similar rate of normal MII than the FFC group (80.4%, *p*=0.1263) and the FFEI/II group (57.5%, *p*=0.5027). The FFEI/II group had lower normal MII rate than No-FF (*p*=0.0380) and FFC (*p*=0.0277).

Therefore, we concluded that samples obtained in 2013 were well preserved and adequate for our experiments because the rates of normal MII were similar to that found in previous work of our group (Da Broi *et al.*, 2014; Giorgi *et al*., 2016). Da Broi *et al*. (2014) found normal MII rate of 76.47% in the No-FF group, 80.85% in the FFC group and 44.44% in the FFEI/II group. Giorgi et al. (2016) found normal MII rate of 86.36% in the No-FF group, 83.52% in the FFC group and 51.35% in the FFEI/II group.
